# Type I Interferons in Bacterial Infections: Taming of Myeloid Cells and Possible Implications for Autoimmunity

**DOI:** 10.3389/fimmu.2014.00431

**Published:** 2014-09-11

**Authors:** Emily M. Eshleman, Laurel L. Lenz

**Affiliations:** ^1^Department of Immunology and Microbiology, University of Colorado School of Medicine, Aurora, CO, USA; ^2^Department of Biomedical Research, National Jewish Health, Denver, CO, USA

**Keywords:** interferons, interferon receptors, bacterial pathogens, macrophage activation, immune suppression, autoimmunity

## Abstract

Type I interferons (IFNs) were first described for their ability to protect the host from viral infections and may also have beneficial effects under specific conditions within some bacterial infections. Yet, these pleiotropic cytokines are now known to exacerbate infections by numerous life-threatening bacteria, including the intracellular pathogens *Listeria monocytogenes* and *Mycobacterium tuberculosis*. The evidence that such detrimental effects occur during bacterial infections in both animals and humans argues for selective pressure. In this review, we summarize the evidence demonstrating a pro-bacterial role for type I IFNs and discuss possible mechanisms that have been proposed to explain such effects. The theme emerges that type I IFNs act to suppress myeloid cell immune responses. The evolutionary conservation of such anti-inflammatory effects, particularly in the context of infections, suggests they may be important for limiting chronic inflammation. Given the effectiveness of type I IFNs in treatment of certain autoimmune diseases, their production may also act to raise the threshold for activation of immune responses to self-antigens.

## Introduction

Type I interferons (IFNs) are a class of cytokines that includes numerous IFNα subtypes, IFNβ, IFNδ, IFNε, IFNκ, IFNτ, and IFNω (1, 2). These secreted factors are predominantly produced by innate immune and non-immune cells of humans and other animals in response to recognition of conserved microbial products, rather than specific antigens. The different type I IFNs vary in their sequences but bind and signal using a common, ubiquitously expressed, heteromeric cell surface receptor (IFNAR) comprised of IFNAR1 and IFNAR2 chains. Ligation of IFNAR in diverse cell types activates a canonical JAK/STAT signaling cascade primarily involving JAK1, Tyk2, STAT1, and STAT2 proteins. Activation of these factors leads to induced transcription of numerous type I IFN-stimulated genes (ISGs), the protein products of which largely act to disrupt various stages of viral replication (3, 4). Type I IFNs are thus important for resistance to several viral infections and are used in the clinic for effective antiviral therapy (2). However, type I IFNs also exert a variety of other effects on cellular functions and immune responses. For example, they up or down regulate production of and responsiveness to other cytokines, chemokines, and can stimulate cell growth, cell survival, or apoptosis (2, 5). Consequently, these cytokines are also used for treatment of melanomas, leukemias, and other cancers (6), and as immune modulatory agents to suppress neuroinflammation in patients suffering from relapse-remitting multiple sclerosis (MS) (2). Hence, type I IFNs exert seemingly opposing pro- or anti-inflammatory effects and pro- or anti-apoptotic effects. It is likely that these opposing effects reflect cell type-specific differences in the activation of secondary or “non-canonical” signaling events and/or variations in the dominance of a specific type I IFN species. Indeed, individual type I IFN proteins vary in their ability to elicit specific responses and stimulation of different cell types can cause distinct signaling events (7, 8).

The second class of IFN protein (type II or IFNγ) is more critical for host defense against intracellular bacterial pathogens, such as *Mycobacterium tuberculosis* and *Listeria monocytogenes*. IFNγ signals through its own ubiquitously expressed heteromeric receptor (IFNGR), which utilizes IFNGR1 and IFNGR2 chains to activate a canonical JAK/STAT pathway primarily involving JAK1, JAK2, and STAT1. In contrast to type I IFNs, which are broadly expressed, IFNγ is produced primarily by lymphocytes. Antigen-specific IFNγ production occurs when appropriate T lymphocyte populations respond to specific microbial antigens, while antigen non-specific production of IFNγ is stimulated by cytokines such as interleukin (IL)-12 and IL-18. Studies using *L. monocytogenes* and other bacterial infection models indicate that both T and natural killer (NK) cells are capable of this antigen non-specific IFNγ production (9–11). Myeloid cells such as macrophages and dendritic cells (DCs) are key targets of IFNγ, as shown by the increased susceptibility to *L. monocytogenes* infection in mice selectively defective for functional IFNGR1 in myeloid cells (12, 13). The expression of numerous IFNγ activated genes (GAGs) is induced by the cytokine. Some of these genes are identical to ISGs and have antiviral effects. However, IFNγ is unique in its ability to elicit a potent anti-microbial state of activation in macrophages. This “M1-type” activation is associated with increased expression of GAGs such as nitric oxide synthase 2 (NOS2) and NADPH oxidase subunits. These enzymes generate nitric oxide (NO) and reactive oxygen species (ROS) that alter cell signaling and under appropriate circumstances can mediate direct killing of bacteria (14, 15). IFNγ-inducible GTPases also promote macrophage resistance to bacterial and parasite infections by increasing the ability of phagosomal compartments to contain and kill engulfed microbes (16–18). IFNγ also upregulates myeloid cell expression of MHC II and other factors important for antigen presentation and T cell activation (19). In addition, IFNγ impacts maintenance and proliferation of hematopoietic stem cells (HSC). Specifically, basal production of IFNγ in the absence of infection drives HSC cycling and the elevated levels occurring during infection can activate HSC proliferation and myelopoiesis to replenish monocytes and other immune cells (20, 21).

Despite the antiviral effects of type I IFNs, and in the context of the antibacterial effects of IFNγ, it is increasingly evident that host responsiveness to type I IFNs correlates with increased host susceptibility to infections by *L. monocytogenes*, *M. tuberculosis*, *Francisella tularensis*, and several other intracellular bacterial pathogens (22–24). Here, relying heavily on the *L. monocytogenes* model, we review the pathways involved in the induction of type I IFNs by intracellular bacteria and various mechanisms proposed to account for the suppressive effects of type I IFN signaling. A theme that emerges from these studies is that type I IFNs have suppressive effects on anti-microbial and antigen-presenting function of myeloid cells. Such effects may contribute to both the observed ability of these cytokines to increase host susceptibility during bacterial infections and to their effectiveness in therapy of neuroinflammatory disease.

## Bacterial Factors Contributing to Type I IFN Production during *L. monocytogenes* Infection

*Listeria monocytogenes* is a Gram-positive facultative intracellular bacterium that causes the systemic disease Listeriosis. The mortality rate of Listeriosis is quite high even in hospitalized patients; hence, *L. monocytogenes* remains a leading cause of death from foodborne illnesses within the United States. *L. monocytogenes* can infect hematopoietic and non-hematopoietic cell types through phagocytosis or cellular mediated uptake (25, 26). Following systemic infection in the murine model *L. monocytogenes* localizes to the liver and spleen where resident phagocytes, primarily macrophages and DCs, engulf the bacteria. *L. monocytogenes* that escape from phagosomal compartments in these cells can replicate within the cell cytosol and further propagate the infection into neighboring cells. To facilitate vacuolar escape, the bacteria secrete a pore-forming hemolysin (hly) known as listeriolysin O (LLO) (27).

Like many other bacteria, *L. monocytogenes* induces production of pro-inflammatory cytokines such as TNFα and type I IFNs when engulfed by professional phagocytes. Studies of infection in bone marrow derived macrophages (BMM) suggest that there are two waves of the cellular response to *L. monocytogenes* infection (28, 29). An “early phase” gene expression profile is seen at 1–2 h post infection by both virulent wild-type *L. monocytogenes* and avirulent Δhly or heat-killed bacteria that cannot escape from vacuolar compartments into the host cytosol (28, 29). Several of these “early phase” genes, including *il1b*, *tnfa*, and those encoding several chemokines are induced through the activation of Toll-like receptor (TLRs) and the ensuing activation of NFκB (28, 29). A subsequent “late-phase” response is observed at 4–8 h after infection by wild-type, but not killed or Δhly, *L. monocytogenes* strains (28, 29). “Late-phase” genes include IFNβ, multiple subtypes of IFNα, and several ISGs (28, 29). The fact that killed and Δhly *L. monocytogenes* strains fail to induce this late-phase IFN-dominated response supports the interpretation that products from bacteria replicating within the BMM cytosol stimulate cytosolic pathogen recognition receptors (PRR), though TLR stimulation can augment the induction of type I IFNs during *L. monocytogenes* infection (29, 30). There has been considerable interest in identifying the cytosolic PRRs responsible for type I IFN production during *L. monocytogenes* infection.

## Pathways Leading to the Production of Type I IFNs during Bacterial Infection

**Figure 1:**
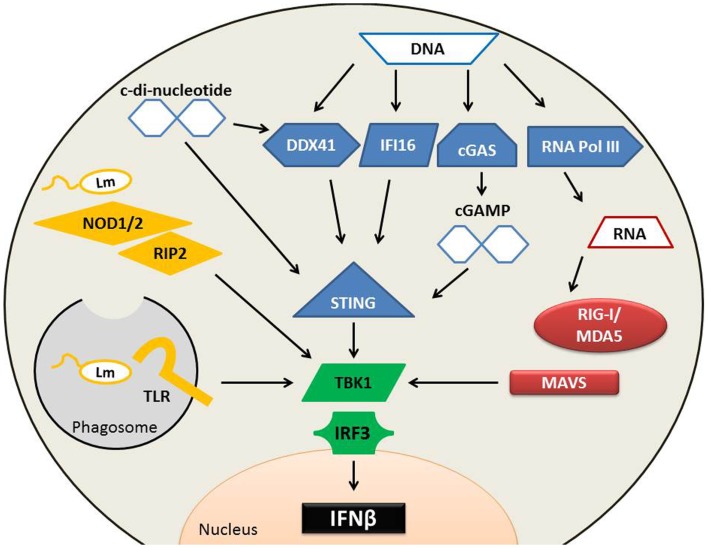
**Pathways implicated in type I IFN production following *L. monocytogenes* infection**. Several cytosolic receptors are able to recognize *L. monocytogenes* (Lm) microbial components to induce type I IFN production. (Yellow) Endosomal TLRs recognize a variety of Lm bacterial patterns including cell wall fragments, which can also stimulate NOD proteins. NOD proteins require association with RIP2 to activate TBK1. Lm secretes RNA, DNA, and cyclic-di-nucleotides. Secreted RNA (red) binds to RIG-I or MDA5, both of which associate with MAVS. Cytosolic DNA (blue) can alternatively be converted into RNA by RNA polymerase III and induce type I IFNs through the RIG-I pathway. DNA is also directly sensed by DDX41 or IFI16 to induce the production of type I IFNs in a STING-dependent mechanism. cGAS can sense DNA and convert it into cGAMP, which binds with high affinity to STING to stimulate IFNβ production. C-di-nucleotides bind to STING directly and to DDX41, either of which may result in IFNβ synthesis. TBK1 and IRF3 are essential for the induction of type I IFN production during Lm infection, and each of these upstream sensing pathways converges on TBK1 activation.

Pathways known to be important for induction of type I IFN within *L. monocytogenes*-infected phagocytes are diagrammed in Figure 1. Amongst the earliest identified cytosolic PRRs were the nucleotide-binding oligomerization domain (NOD)-containing proteins; members of the nucleotide-binding domain, leucine-rich repeat (LRR) protein family referred to as NLRs. NOD1 and NOD2 proteins sense distinct muropeptide fragments from the cell wall from *L. monocytogenes* and other bacteria (31–33). Recognition of appropriate muropeptides activates a serine/threonine kinase receptor interacting protein (RIP2) to initiate downstream signaling and activation of NFκB (31). With regards to triggering of type I IFN production, NOD1, NOD2, and RIP2 seem to play an ancillary role. They augment the induction of type I IFN and other cytokine expression in response to *L. monocytogenes* (29, 31–33), but mice and BMM deficient for any one of these proteins retain the ability to mount inflammatory responses and synthesize type I IFNs in response to *L. monocytogenes* (31, 34, 35). Thus, the recognition of bacterial cell wall components by the cytosolic NOD1 and NOD2 proteins is not crucial for the induction of type I IFNs during *L. monocytogenes* infection.

Nucleic acids are potent inducers of type I IFN production and it was shown that extracts from *L. monocytogenes* induce IFNβ production in a manner sensitive to DNAse treatment of the extracts (36). *L. monocytogenes* was also reported to actively secrete both RNA and DNA during infection of macrophages (37). Cytosolic RNA is detected by the RNA helicase retinoic acid inducible gene 1 protein (RIG-I), related RIG-I-like (RLR) proteins including melanoma differentiation-associated gene 5 (MDA5), as well as other non-RLR helicases and PRRs (38–40). RNA recognition by RIG-I and MDA5 induces their recruitment to mitochondria, where they encounter an adaptor protein [mitochondrial antiviral signaling (MAVS)] that regulates downstream signaling to induce type I IFNs (41–43). Secreted *L. monocytogenes* DNA could also be detected using these RNA receptor systems if it is transcribed into RNA by host cell RNA polymerase III (37). However, deficiency in RIG-I, MDA5, or MAVS fails to ablate IFNβ production by *L. monocytogenes*-infected BMM (37, 44, 45). Thus, it does not appear that RNA sensing is crucial for recognizing cytosolic *L. monocytogenes* infection in this cell type, although it may play a more important role in sensing *L. monocytogenes* infection of other cell types and for sensing infection by other bacteria, namely *Legionella pneumophila* (46, 47).

DNA present in the host cell cytosol can also be detected and trigger the production of type I IFNs (40). Several putative receptors have been identified that might mediate such recognition, including the DNA-dependent activator of IRFs (DAI), IFN-inducible gene (IFI)-16, and LRR flightless-interacting protein (LRRFIP1). Deficiency or knockdown of DAI, IFI16, or LRRFIP1 fails to completely ablate type I IFN production by infected murine BMM (48–50). However, recently IFI16 was shown to have a larger role in the recognition of *L. monocytogenes* DNA in the human macrophage cell line, THP1. Knockdown of IFI16 in these cells drastically reduced the production of IFNβ in response to *L. monocytogenes* DNA (51). The DNA-binding DEAD-box helicase DDX41 has also been shown to bind *L. monocytogenes* DNA. DDX41 elicits type I IFN production through a mechanism requiring the stimulator of interferon genes protein (STING), also called MITA, MPYS, or ERIS (52). STING induces type I IFN production by activating the TNFR-associated NF-κB kinase (TANK)-binding kinase 1 (TBK1), which phosphorylates a C-terminal serine residue on the transcription factor IFN regulatory factor 3 (IRF3) to induce IRF3 dimerization and nuclear translocation (34, 53). IRF3 and other IRF family members bind to the promoters of type I IFN genes and ISGs to regulate and initiate their transcription. TBK1 and IRF3 are thus not surprisingly essential for the production of type I IFNs during *L. monocytogenes* infection (34, 54). Likewise, deficiency or knockdown of STING significantly decreases IRF3 activation and IFNβ production in BMM, DC, or fibroblasts infected with *L. monocytogenes* (55–57).

It appears that STING does not respond to intact DNA, but rather to endogenous or exogenous cyclic-di-nucleotides (40). In the presence of cytosolic dsDNA, the enzyme cGAS synthesizes an endogenous cyclic-di-nucleotide, cGAMP (58). Binding of cGAMP to STING occurs with a very high affinity (~4 nM) and induced conformational changes that presumably initiate the downstream events that culminate in type I IFN production (59). Exogenous cyclic-di-nucleotides can also activate STING (56, 57, 60). Both cyclic-di-GMP (cdGMP) and cyclic-di-AMP (cdAMP) are produced by bacteria and function as second messengers. There is evidence that cdAMP is released from replicating *L. monocytogenes* (61, 62), thus it is conceivable that cdAMP secreted by the bacterium mediates the STING-dependent production of type I IFNs in *L. monocytogenes*-infected macrophages. However, while the affinity of cdAMP for STING is not known, STING binds cdGMP ~300-fold lower affinity (~1 μM) than cGAMP DNA (59). Thus, it is also conceivable that cGAMP produced by cGAS in response to secreted bacterial DNA contributes to STING-dependent type I IFN production. Regardless, it is important to keep in mind that STING deficient mice showed significantly reduced serum IFNβ only very early (8 h) after *L. monocytogenes* infection (56, 57). Thus, systemic *L. monocytogenes* infection can trigger type I IFN through multiple pathways and the impact of STING on overall type I IFN production in this model is limited.

## Type I IFN Signaling and Increased Susceptibility to Bacterial Infection

In certain bacterial infection models, protective effects of type I IFNs have been reported. For example, type I IFN can reduce bacterial burdens in cultured cells infected with *L. pneumophila* or *Chlamydia trachomatis* and survival of mice is increased in sepsis models with group B *Streptococcus* and *E. coli* (63–65). Mice lacking expression of IFNε, which is abundantly expressed within the female reproductive tract, were also reported to be highly susceptible to urogenital infection by *C. muridarum* (66). The precise mechanisms are not clear in these cases, but the observed protective effects appear to reflect unique aspects of the models and/or pathogens studied since there is considerable evidence to indicate that type I IFNs instead play a deleterious role during infections by numerous other bacterial pathogens (22–24). Specifically, studies with mice lacking IFNAR1 report that bacterial burdens are significantly reduced and survival increased following systemic or mucosal infections with intracellular bacteria that infect the cytosol of host cells, such as *L. monocytogenes* (54, 67–70) and *F. tularensis* (71) as well as bacteria like *M. tuberculosis* (72–74) and *C. muridarum* (75, 76) that reside within vacuolar compartments. In addition, heightened type I IFN production correlates with increased host susceptibility to several bacterial infections. In mice, examples of this include the correlation of increased type I IFN production in mice with a mutated ubiquitin specific peptidase (USP18) and sensitivity to *Salmonella typhimurium* (77). Furthermore, isolates of *L. monocytogenes* and *M. tuberculosis* that hyper-induce type I IFN production have heightened pathogenicity in animal models (78, 79). The administration of type I IFNs or agents that induce these cytokines also causes increased susceptibility to *L. monocytogenes* and *M. tuberculosis* in model infections (54, 78, 80). Type I IFN production is also increased during viral infections. In mice, lymphocytic choriomeningitis virus (LCMV) infection potently induces type I IFNs and leads to ~1000-fold increased susceptibility to a secondary *L. monocytogenes* infection as measured by bacterial burdens (81). In humans, a similar situation occurs following infection with influenza virus. Influenza infections are often associated with secondary bacterial infections and secondary bacterial pneumonias are estimated to account for up to 25% of the more than 250,000 annual deaths attributed to influenza (82, 83). Such secondary infections are also thought to have caused most of the deaths from the 1918 influenza pandemic (84). *Streptococcus pneumoniae* is a prevalent bacterial cause of pneumonias and a model of influenza and secondary *S. pneumoniae* infection showed that increased bacterial burdens and mortality was dependent on IFNAR expression (85). Severe bacterial infections have also been noted in patients receiving prolonged IFNα2 therapy for chronic hepatitis C virus infection (86–88). Moreover, in the absence of obvious viral infections, signatures of type I IFN responses correlate with disease progression in human tuberculosis and leprosy patients (89, 90). Thus, despite numerous differences in the receptors and cytokines themselves, the association of type I IFNs with exacerbated bacterial infections appears to have been conserved in murine and human systems. An improved understanding how these cytokine responses are deleterious to their hosts and what has driven their conservation across this evolutionary span are important questions to address.

## Mechanisms Proposed to Account for the Pro-Bacterial Effects of Type I IFNs

**Figure 2:**
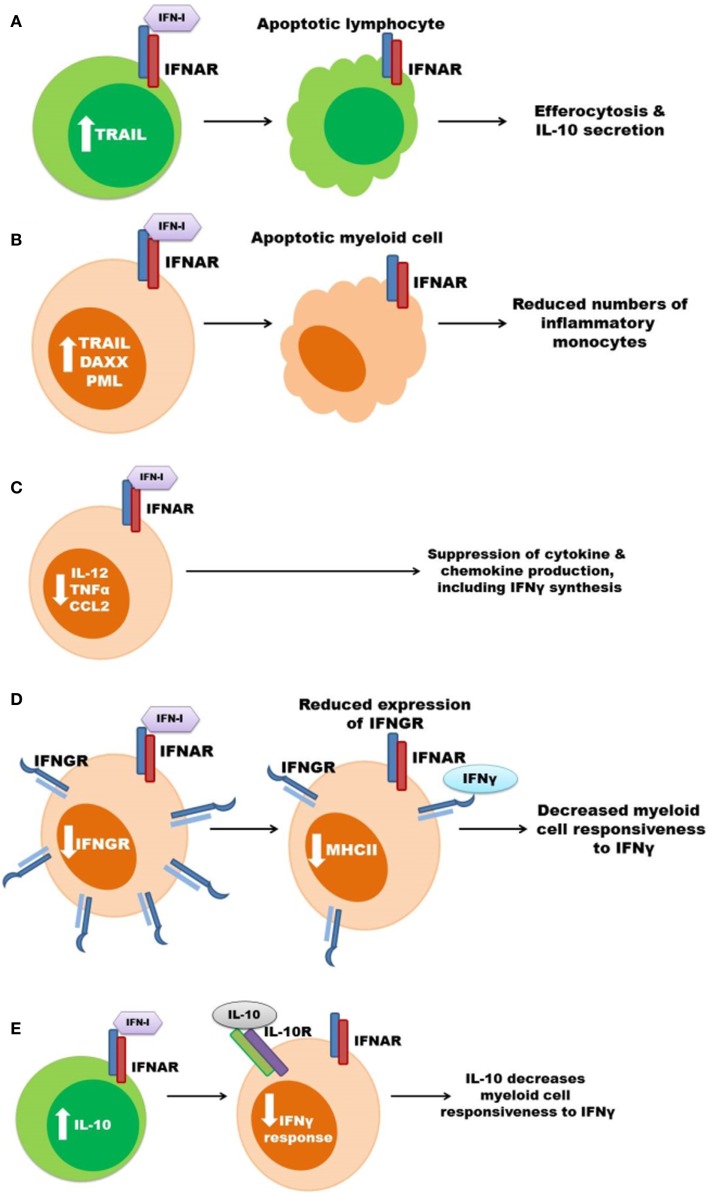
**Some mechanisms previously proposed to account for the pro-bacterial effects of type I IFNs**. **(A)** Type I IFNs up regulate pro-apoptotic genes resulting in lymphocyte (green) apoptosis. Apoptotic lymphocytes stimulate myeloid cell IL-10 secretion. **(B)** Increased apoptosis of myeloid cells (orange) leads to reduced amounts of inflammatory monocytes during infection. **(C)** Signaling through the IFNAR suppresses myeloid cell secretion of pro-inflammatory cytokines and chemokines, which can result in decreased IFNγ production. **(D)** Expression of IFNGR is suppressed in response to type I IFN signaling in myeloid cells thus decreasing cellular responsiveness to IFNγ. **(E)** Type I IFNs induce the production of IL-10 to inhibit IFNγ responsiveness.

A summary of the proposed mechanisms for the deleterious effects of type I IFN signaling during bacterial infections is outlined in Figure 2.

### Induction of host cell death

It has long been known that bacterial infections can induce death of multiple cell types within tissues of murine hosts. In the systemic *L. monocytogenes* infection model, this cell death is exacerbated by type I IFNs. O’Connell et al. observed that expression of pro-apoptotic genes such as TNF-related apoptosis-inducing ligand (TRAIL), promyelocytic leukemia (PML), and death-associated protein 6 (Daxx) were increased in the spleens of *L. monocytogenes*-infected wild-type, but not IFNAR1 deficient, mice (54). Consistent with increased apoptosis in these tissues, terminal deoxynucleotidyl transferase-mediated dUTP nick-end labeling (TUNEL) staining is also increased in the spleens of infected wild-type mice, when compared to infected IFNAR1^−/−^ or IRF3^−/−^ mice (54, 69). This TUNEL staining was localized to lymphocyte rich follicles within the spleens, suggesting that type I IFN might induce apoptosis of lymphocytes and that such apoptosis could itself be detrimental (54, 69). The possibility that lymphocyte apoptosis is deleterious to the host is also consistent with the observation that mice deficient in lymphocytes or T cells alone are resistant to acute systemic *L. monocytogenes* infection (91–93). Resistance in T cell-deficient hosts is thought to reflect constitutively heightened macrophage activation (94), and correlates with reduced production of the anti-inflammatory cytokine IL-10 (91). It was thus proposed that in mice responsive to type I IFNs, the uptake of apoptotic cells by macrophages triggers their production of IL-10, which in turn inhibits host resistance (91). Myeloid cells are also sensitive to apoptosis in response to type I IFN and IFNAR expression has also been correlated with increased apoptosis of splenic and pulmonary macrophages during *L. monocytogenes* and pulmonary *C*. *muridarum* infections, respectively (54, 76). As mentioned above, these type I IFNs increase expression of the pro-apoptotic factor TRAIL during *L. monocytogenes* infection (54). Similar to IFNAR1^−/−^ mice, mice lacking TRAIL demonstrate reduced TUNEL staining and increased resistance during *L. monocytogenes* infection (95). These effects were further correlated with increased numbers of splenic lymphocytes and monocytes. Type I IFN production induced by LCMV infection also correlates with granulocyte apoptosis and impaired control of *L. monocytogenes* infection (81). Thus, there is a clear association between type I IFNs, cellular death, and impaired myeloid cell responses during bacterial infections. Nonetheless, it remains unclear whether apoptosis of T or myeloid cells is a primary cause of the increased host susceptibility.

### Suppression of pro-inflammatory cytokine and chemokine production

Type I IFN production during viral infection is known to suppress production of IL-12 and other pro-inflammatory cytokines (96). Similarly, type I IFN production was associated with reduced secretion of IL-12 and TNFα in both *L. monocytogenes* and *M. tuberculosis* infection models (68, 72). Type I IFNs also suppress IL-1β production by inhibiting inflammasome activation (97), and reduced IL-1β secretion correlated with increased host susceptibility in *M. tuberculosis* infection models (98, 99). Expression of chemokines such as CCL2 is also regulated by type I IFNs (100, 101). CCL2 and its chemokine receptor CCR2 are critical for migration of inflammatory monocytes to sites of infection by *L. monocytogenes* and other bacteria (102–104). Spleens of IFNAR1^−/−^ mice have increased accumulation of inflammatory monocytes during *L. monocytogenes* infection (68), however, type I IFNs upregulate CCL2 and recruitment of monocytes into the lung during *M. tuberculosis* infection (80). In the latter study, accumulation of monocytes correlated with more severe infection. By contrast, type I IFNs were reported to impair production of CXCL1, CXCL2, and neutrophil accumulation in lungs and more severe infection in mice infected with *S. pneumoniae* (85). Moderately, impaired neutrophil recruitment was also correlated with reduced IL-17 production and increased disease severity in mice in response to type I IFNs during *F. tularensis* and *L. monocytogenes* infections (71). However, it is debated whether or not neutrophils are protective during infections by *L. monocytogenes* and other intracellular bacteria (105, 106). Moreover, the neutropenia seen in patients treated with type I IFNs fails to correlate with their susceptibility to bacterial infections (86, 87). Thus, type I IFNs can alter production of cytokines and chemokines involved in neutrophil or inflammatory macrophage recruitment.

### Suppression of myeloid cell responsiveness to IFNγ

IFNγ is critical for the pro-inflammatory anti-microbial (M1) type activation of macrophages and transgenic mice lacking responsiveness to IFNγ selectively in myeloid cells are highly susceptible to *L. monocytogenes* and other intracellular pathogens (12, 13, 107). The macrophages activated by IFNγ have increased expression of molecules involved in both MHC class I and MHC class II antigen presentation, as well as enzymes producing reactive oxygen and nitrogen species with potential anti-microbial functions and pro-inflammatory cytokines and chemokines, including IL-12 (19). Expression of some, but not all, of these genes can also be induced when macrophages are stimulated with type I IFNs. In contrast, only IFNγ stimulates macrophages to express or upregulate MHC class II molecules (108). Indeed, stimulation of macrophages with type I IFNs suppresses their induction of MHC II expression in response to IFNγ. As mentioned above, type I IFNs also suppress production of IL-12 and CXCL1 and 2 (85, 96). These data suggest that type I IFNs are able to prevent or dampen classical M1-type anti-microbial macrophage activation in response to IFNγ. Consistent with this interpretation, a recent report revealed an inverse correlation between IFNβ and IFNγ gene expression patterns in lesions of human leprosy patients (90). The IFNβ-driven response also correlated with IL-10 production, and IFNβ production contributed to IL-10 secretion, leading the authors to conclude that the impaired IFNγ responses in *M. leprae* infected macrophages is due to IL-10 production (90). Indeed, IFNβ and IL-10 treatments both impaired the ability of IFNγ to induce expression of the vitamin D receptor, the vitamin D-1a-hydroxylase, and the anti-microbial peptides cathelicidin and DEFB4 in macrophages (90).

Type I IFNs were also associated with the induction of IL-10 secretion and Programed death-ligand 1 (PD-L1) expression by myeloid cells during chronic LCMV infection (109, 110). Experiments using antibody blockade of IFNAR showed reduced expression of these immune suppressive factors and increased clearance of persistent viral infections (109, 110). Interestingly, the blockade of IFNAR also suppressed production of IL-1β and IL-18, arguing against the notion that improved viral clearance was due to increased inflammasome activation. Rather, the improved viral clearance was associated with increased serum IFNγ and blockade of IFNAR failed to improve viral clearance in mice treated with antibody to block IFNγ (109, 110). The therapeutic effects of blocking type I IFN signaling also correlated with an improved ratio of stimulatory versus immune regulatory antigen-presenting cells (APCs) and enhanced antiviral T cell responses. Although the authors of one study further suggested that the suppression of inflammatory and immune responses in these studies reflected chronic type I IFN signaling (109), a second study observed that type I IFNs increased IL-10 secretion and PD-L1 expression by DCs as early as 1 day post infection (110).

Leading up to these recent studies, prior efforts had also demonstrated suppressive effects of type I IFNs on myeloid cell activation during systemic *L. monocytogenes* infection of mice (70). In this model, the suppressive effects of type I IFNs correlate with reductions in myeloid cell surface IFNGR1. Similarly, surface IFNGR1 staining is significantly reduced on myeloid cells from *M. tuberculosis* infected patients compared to healthy control and effective treatment of these patients correlates with restored myeloid surface expression of IFNGR1 (111). IFNAR expression is necessary and recombinant type I IFNs are sufficient to trigger IFNGR1 down regulation in mouse and human myeloid, but not T cells (70, 112), suggesting this mechanism might contribute to a selective inhibition of myeloid cell responsiveness to IFNγ. Indeed, the reduced expression of IFNGR1 correlates with decreased responsiveness to IFNγ as indicated by reduced STAT1 phosphorylation and impaired induction of MHC class II expression in the context of *L. monocytogenes*, *M. tuberculosis*, and *F. novicida* infections (70, 111, 113).

Additional mechanistic studies have revealed that type I IFNs suppress myeloid cell surface IFNGR1 within hours of stimulating the IFNAR and that this effect is due to transcriptional silencing of the otherwise constitutively expressed *ifngr1* gene (70, 111, 112). The rapid reductions in *ifngr1* transcript abundance following IFNβ stimulation are preceded by loss of activated RNA polymerase II at the *ifngr1* transcriptional start site and the accumulation of epigenetic marks on nearby histones that are indicative of condensed chromatin (112). The reduction in *ifngr1* transcription is also associated with recruitment of the early growth response 3 (Egr3) transcription factor shortly after IFNβ treatment (112). Egr3 is a member of the Egr family of zinc finger transcription factors originally defined for their role in regulation of cell growth and differentiation (114, 115). Egr3 can act as an activator or repressor in response to various stimuli, depending on post-translational modifications and association with various adapter proteins (114–117). One such adaptor protein, the NGFI-A binding protein Nab1 is a known corepressor and is also recruited to the *ifngr1* promoter shortly after Egr3 (112). Knockdown of Nab1, but not Nab2, prevented IFNGR1 down regulation in macrophages treated with IFNβ, suggesting that recruitment of a repressive Egr3/Nab1 complex is responsible for rapid silencing of *ifngr1* transcription (112). Given that the half-life of IFNGR1 protein is estimated at 3–4 h (118), such transcriptional silencing is sufficient to rapidly reduce myeloid cell responsiveness to IFNγ. Nonetheless, type I IFN stimulation does not appear to cause a complete loss of myeloid cell surface IFNGR1, possibly due to the induction of SOCS proteins and other endogenous negative feedback circuits that attenuate cellular responses to IFNAR signaling. These results suggest that down regulation of IFNGR1 expression might be an early step in the cascade of events leading to the suppression of myeloid cell responses that result in increased bacterial burdens and disease severity during acute and chronic bacterial infections, and the establishment or maintenance of chronic viral infections.

### Common features of proposed mechanisms

Given the numerous effects of type I IFNs on various cells of the immune system and on non-immune cells, it is plausible that their pro-pathogen effects vary for different pathogens. This seems particularly likely for pathogens infect different tissue or cell types, where the responses to type I IFNs may differ. For instance, a recent study demonstrated that IFNAR expression on non-hematopoietic cells was required to increase host susceptibility to the intracellular bacterial pathogen *Ixodes ovatus Ehrlichia* (119). The effects of these cytokines may also differ depending on the route of infection and the presence or nature of competing commensal microbes. For example, Kerbauer et al. suggested that type I IFNs might not be as detrimental to the host following gastric infection of mice with *L. monocytogenes* (120). Regardless, results from the studies highlighted above clearly implicate myeloid cells/APCs as key targets of type I IFNs in settings where these cytokines are deleterious to the host. Precisely, how these cytokines act to dampen myeloid cell immunity and what selective advantage this confers on the host remains to be discerned.

## Apposing Effects of Type I IFNs in Autoimmune Diseases

The role of type I IFN signaling during autoimmune disease remains controversial, possibly indicating that these cytokines have opposing effects in different disease settings. For example, chronic type I IFN production is a hallmark of systemic lupus erythematosus (SLE) and several groups have reported a subset of ISGs upregulated in SLE patients compared to healthy controls (121–124). ISG signatures were also associated with disease severity and progression in SLE patients (121–123). It has thus been suggested that type I IFNs promote SLE pathology through the activation of effector cells. Type I IFNs can paradoxically promote not just death of lymphocytes, but also T cell survival, proliferation, cytotoxicity, and B cell differentiation and antibody production. Any of these effects might conceivably contribute to increased tissue damage and disease progression in SLE patients.

In contrast to the exacerbation of SLE by type I IFNs, these same cytokines confer therapeutic benefits in certain other autoimmune diseases. The most obvious example of this is the neuroinflammatory disease MS. IFNβ is a common therapy and has been shown to reduce the frequency of clinical exacerbations in patients with relapse-remitting MS (125). The mechanisms for these beneficial effects remain uncertain. However, type I IFNs also suppress disease in the murine experimental autoimmune encephalomyelitis (EAE) model of MS. As for MS, IFNβ is therapeutic in the EAE model and deletion of the *ifnb* gene or IFNAR1 robustly increased EAE pathogenesis in mice (126, 127). Furthermore, using conditional knockouts, it was shown that IFNAR1 expression on myeloid cells was specifically required for the therapeutic effects of IFNβ during EAE (127). Deficiencies in IFNAR1 expression on myeloid cells also severely exacerbated disease and correlated with increased secretion of TNFα and CCL2 as well as increased expression of MHC II (127). These immunosuppressive effects of IFNβ treatment in humans may likewise target myeloid cells.

Other autoimmune diseases where type I IFNs appear to play a protective role include collagen type II induced arthritis in non-human primates (128). Treatment with double-stranded RNA species or recombinant IFNα also lowered the frequency and severity of arthritic symptoms in the murine model of antigen-induced arthritis (129). The pharmacokinetics of IFNβ therapies have shown to be a barrier is translating many of these treatments from animal models to clinical use in humans. However, Mullen et al. engineered a latent form of IFNβ that can only become activated when cleaved by aggrecanase (130). Aggrecanases are highly expressed within the joints and synovial fluid of rheumatoid arthritis and osteoarthritis patients and are responsible for the cleavage of aggrecan, an important component of joint tissue (130). This delivery method allows for temporal and tissue specific release of IFNβ that resulted in a significantly increased half-life of IFNβ as well as reduced pathology and joint swelling from collagen induced arthritis (130).

Humans with autoimmunity often carry a single nucleotide mutation in protein tyrosine phosphatase non-receptor type 22 (PTPN22) (131). PTPN22 is an intracellular protein tyrosine phosphatase that is exclusively found in immune cells (131), and was recently associated with TLR signaling for type I IFN synthesis in myeloid cells (132). Functional PTPN22 was also shown to suppress inflammatory arthritis and promote gut homeostasis (132). Mice deficient in PTPN22 demonstrate increased susceptibility in the dextran sodium sulfate (DSS) mouse model of acute colitis (132). TLR stimulation by microbiota also induced immunosuppressive effects that correlated with type I IFN production and decreased progression of experimental colitis in mice (133). Treatment with recombinant IFNβ phenocopied the decreased colitis achieved through TLR stimulation (133). Moreover, in a randomized placebo controlled study of active ulcerative colitis, a significant clinical response, and in some cases, disease remission, was seen in patients that received IFNβ therapy compared to the patients that received placebo (134, 135). It was noted that the therapeutic effects of IFNβ treatment in this disease correlated with reduced production of IL-13, an effector cytokine driving intestinal inflammation (134). Mice deficient in type I IFN signaling have been shown to have exacerbated DSS-induced acute colitis (136, 137). Furthermore, mice with IFNAR1 deletion specifically in myeloid cells demonstrated significantly increased weight loss and colitis disease activity score when treated with DSS (136). These data suggest that type I IFN signaling specifically in myeloid cell is protective during DSS-induced acute colitis. Interestingly, the authors further showed that IFNAR1^−/−^ mice recovered from DSS treatment more quickly than wild-type mice, suggesting a deleterious role of type I IFNs during the recovery phase of colitis (136).

## Conclusion

Interferons are important mediators and regulators of the immune response to viruses, bacteria, and other pathogens. They can suppress inflammatory responses and exacerbate the pathogenesis in certain autoimmune diseases and several intracellular bacterial infections. Indeed, pathogens such as *L. monocytogenes* may actively promote type I IFN production through secretion of nucleic acids or cyclic-di-nucleotides that are recognized by cytosolic pattern recognition receptors to stimulate a type I IFN response. However, type I IFNs appear to be protective in certain other bacterial infections and in many viral infections, and may exacerbate the autoimmune disease SLE. Thus, blindly blocking their production as a therapy for bacterial infections would likely have severe untoward effects in these other disease settings. It thus remains an important challenge to dissect the mechanisms for the divergent pro- and anti-inflammatory effects of type I IFNs, as well as their paradoxical protective and deleterious effects during infectious and other diseases. As we review here, a number of observations have been correlated with the pro-bacterial effects of type I IFNs. However, while these observations have led to the proposal of several differing mechanisms to explain these detrimental effects of type I IFNs during intracellular bacterial infections, a common theme is the suppression of myeloid cell inflammatory responses. Whether such suppression results from the induction of effector cell death and IL-10 production, suppression of T cell cytokine or chemokine production, suppression of inflammasomes, or down modulation of IFNGR expression remains to be seen. However, even in the absence of experimental proof that points to a specific mechanism, it is attractive to speculate that the deleterious effects of type I IFN signaling during bacterial infections are tolerated because their ability to suppress myeloid cell responses also has a beneficial effect in protecting the host from MS and other autoimmune diseases.

## Conflict of Interest Statement

Laurel L. Lenz serves on editorial boards for Frontiers in Immunology and Frontiers in Microbiology. The authors declare that the research was conducted in the absence of any commercial or financial relationships that could be construed as a potential conflict of interest.
